# Hair salons as a promising space to provide HIV and sexual and reproductive health services for young women in Lesotho: a citizen scientist mixed-methods study

**DOI:** 10.1186/s12889-025-23435-3

**Published:** 2025-07-02

**Authors:** Malena Chiaborelli, Mamaswatsi Kopeka, Pontšo Sekhesa, Madeleine Sehrt, Tsepang Mohloanyane, Tala Ballouz, Dominik Menges, Jennifer A. Brown, Jennifer M. Belus, Felix Gerber, Fabian Raeber, Andréa Williams, David Jackson-Perry, Meri Hyöky, Donaldson F. Conserve, Karen Hampanda, Alain Amstutz, and the Hair SALON Citizen Scientist Working Group, Itumeleng Mohale, Thabo Lebakeng, Masia Morena-motšo, Thembekile Mokhosi Mapefane

**Affiliations:** 1 Division of Clinical Epidemiology, Department of Clinical Research, University Hospital Basel and University of Basel, Basel, Switzerland; 2 Swiss Tropical and Public Health Institute, Allschwil, Switzerland; 3 Department of Prevention and Community Health, Milken Institute School of Public Health, The George Washington University, Washington, DC USA; 4 The HUB, Morija, Lesotho; 5 Colorado School of Public Health, University of Colorado, Denver, USA; 6 Central University of Technology, Bloemfontein, South Africa; 7 Epidemiology, Biostatistics and Prevention Institute, University of Zurich, Zurich, Switzerland; 8 Department of Medical Epidemiology and Biostatistics (MEB), Karolinska Institutet (KI), Stockholm, Sweden; 9 Centre for the AIDS Programme of Research in South Africa (CAPRISA), University of KwaZulu-Natal, Durban, South Africa; 10 Nuffield Department of Primary Care Health Sciences, University of Oxford, Oxford, UK; 11 Infectious Diseases Service, Lausanne University Hospital, Lausanne, Switzerland; 12 School of Social Sciences, Education and Social Work, Queen’s University, Belfast, UK; 13 Department of Obstetrics and Gynecology, University of Colorado Anschutz Medical Campus, Denver, USA; 14 Oslo Center for Biostatistics and Epidemiology, Oslo University Hospital, Oslo, Norway; 15 Bristol Medical School, Population Health Sciences, University of Bristol, Bristol, UK

**Keywords:** HIV, Young women, Sexual and reproductive health, Hair salon, Citizen science, Lesotho

## Abstract

**Introduction:**

Adolescent girls and young women in southern Africa are disproportionately affected by HIV and sexual and reproductive health (SRH) challenges. There is a need for more accessible and de-medicalized community spaces to offer HIV/SRH services for this key population. We aimed to assess the acceptability and feasibility of offering HIV/SRH services at hair salons in Lesotho.

**Methods:**

We used an innovative citizen scientist mixed-methods approach, whereby hair stylists were recruited through social media, completed questionnaires, and recruited women clients aged 15–35 years as respondents. A stepwise verification process including GPS, pictures, and a local mobile payment system ensured data quality. Subsequently, we conducted individual in-depth interviews among 14 stylists and clients, following the rapid thematic analysis framework, supported by natural language processing. Clients and stylists were involved at the design, implementation, and results interpretation stage.

**Results:**

We recruited 157 hair stylists (median age 29; [interquartile range 25–33]; across all ten districts of Lesotho) and 308 women clients (median age 26 [22–30]). Among stylists, 93.6% were comfortable offering oral HIV self-testing (HIVST), 92.4% pre-exposure prophylaxis (PrEP), and 91.7% post-exposure prophylaxis (PEP). Among clients, 93.5%, 88.3%, and 86.4% felt comfortable receiving the above-mentioned services, respectively. Immediate demand for the three services was 30.8%, 22.1%, and 14.9%. Acceptability and demand were higher for family planning methods and menstrual health products. 90.4% of stylists thought that offering HIV/SRH services would positively impact their business. The majority of clients visit their salon once or twice a month. Salons were more accessible than the nearest health facility in terms of cost and time, but only 21.0% have an additional confidential space. Qualitative analysis confirmed high acceptability of hair salons as an accessible, less judgemental space than clinics, but raised concerns regarding confidentiality and stylists’ roles.

**Conclusions:**

This study suggests that offering HIV/SRH services in hair salons in Lesotho seems to be largely acceptable and feasible with some addressable barriers, based on survey data. A pilot intervention, guided by this study’s recommendations, is warranted to translate these findings into practice.

**Supplementary Information:**

The online version contains supplementary material available at 10.1186/s12889-025-23435-3.

## Introduction

Adolescent girls and young women in southern and eastern Africa are at the epicenter of the HIV pandemic due to a number of biological, social, and structural factors [1]. In 2023, they accounted for 63% of all new global HIV acquisitions with more than 4000 acquiring HIV every week [2]. As in other settings with a high HIV prevalence in southern Africa, HIV and pregnancy-related complications are the leading causes of death among this population in Lesotho [3]. Nearly a quarter of the adult population in the country lives with HIV and two-thirds of new HIV acquisitions are among young women [4, 5]. Similarly, high rates of other sexually transmitted infections and unintended pregnancies have been reported in this key population [6, 7].

In 2016, the Lesotho Ministry of Health introduced oral pre-exposure prophylaxis (PrEP) as a prevention strategy into the national HIV program, and as of 2020, promotes broad eligibility criteria with young women being a key population [8, 9]. Despite significant investment in HIV programmes across the region, uptake and continuation of PrEP among this population has been challenging [10]. Access to traditional health facilities is a major challenge with 70% of Basotho living in rural areas [11] and HIV services poorly integrated with other sexual and reproductive health (SRH) services, despite recommendations by the World Health Organization (WHO) [12]. Therefore, innovative community-based integrated HIV/SRH care models are needed to address access barriers and expand HIV/SRH services.

Hair salons may offer a novel and accessible community space to engage young women and address some barriers related to SRH. Women, across all levels of society, spend substantial time in hair salons without male partners, during which they focus on health- and beauty-related issues, receive community news, and socialize. Stylists are often trusted members of the community [13–15]. While there is promising evidence from the United States that hair salons can improve access to certain health services [16–21], evidence from Africa is limited to four small-scale published surveys and qualitative analyses from Durban, South Africa [13–15, 22].

Citizen science, i.e., involving citizens at various research stages, originates from environmental health research [23] with the aim to foster a deeper public engagement with science, tailor the research agenda to the needs of the public, and democratize science. This approach is becoming increasingly important across various research fields [24–26], including HIV implementation science [27].

We conducted a mixed-methods study among hair salon stylists and women clients to assess the acceptability and feasibility of offering HIV/SRH services in salons, applying a citizen science approach in Lesotho, where this community-based model had not been previously explored.

## Methods

### Study design and setting

This was an explanatory sequential mixed-methods study [28] with an emphasis on the quantitative component, which was used to inform the development of the qualitative component. The quantitative component consisted of an online nationwide cross-sectional survey administered between March 2024 and July 2024. The qualitative part entailed in-depth individual interviews, conducted between May 2024 to July 2024, following a rapid thematic analysis approach [29]. The study protocol was registered [30]. We report the study according to the Mixed Method Reporting in Rehabilitation & Health Services (MMR-RHS) checklist [28] (Text S1) and the community and citizen scientist involvement according to the Guidance for Reporting Involvement of Patients and the Public (GRIPP2) checklist [31] (Text S2).

### Citizen-scientist survey

For the survey, we first recruited citizen scientists, i.e., hair stylists across the entire country, using social media and a dedicated registration webpage (https://hairsalonproject.com/). Inclusion criteria for stylists were being aged 18 years or older, working at a women's hair salon in Lesotho (not house call service only), owning an Android smartphone with a functional camera and GPS, using WhatsApp, and having an M-PESA account (a widely used mobile money service in Lesotho). Once successfully registered (i.e., eligibility checked, existence of the hair salon verified, and electronic consent given), the citizen scientists received a link to their questionnaire via WhatsApp and were subsequently asked to recruit their clients. Inclusion criteria for clients were being 15 to 35 years old, self-identifying as women, being clients of the participating stylists, owning an Android smartphone with a camera and GPS, using WhatsApp, and having an M-PESA mobile account. Stylists provided their recruited clients with a link to the same registration webpage. After successful registration on the webpage and providing electronic consent, the clients received their questionnaire via WhatsApp. The data was centrally verified by the study coordinator in real-time, through a stepwise process, including checks for GPS location, address matching, duplicate detection (via name, phone number, and M-Pesa account), and photo review. Inconsistencies were resolved through direct phone contact when needed (details in Figure S1).

To quantitatively assess the acceptability of offering/receiving HIV/SRH services among stylists/clients, we used three different measures. First, we asked stylists and clients to rate their level of comfortability of offering/receiving thirteen different HIV/SRH services (Text S3) on a 5-level likert-scale (strongly disagree to strongly agree). Second, we asked the clients which of the thirteen services they would be interested in receiving if provided at the hair salon within the coming 6 months. Third, we asked the stylists if they believed providing any of these services would have a positive/negative impact on their business (questions on a 5-level likert-scale). Feasibility considerations in the stylists’ questionnaire encompassed the salon’s infrastructure, opening hours, staff and client volume, as well as reimbursement expectations. The clients’ feasibility questionnaire section entailed the frequency, duration and resource use of a typical salon visit versus a typical health facility visit. Among all participants, we collected socio-demographic data, awareness of HIV self-testing (HIVST) and PrEP, PrEP myths and perceptions (adapted from [32]), age of menarche, menstrual material use [33], impact of menstruation on work/school attendance, current family planning methods use and supply location, and clients’ own perceived HIV risk (one-question risk index from prior research [34]). We collaboratively developed the questionnaires in English and Sesotho and piloted them during a citizen scientist workshop (Text S2). The questionnaires are available in the supplement (Text S4 and S5). The questionnaires were deployed through the web interface of KoboCollect [35, 36], transferring the data via a secured channel onto a password-protected server. Using an application programming interface via KoboconnectR [37] and a project dashboard based on shinyR [38], the study coordinator monitored and verified the data real-time.

### Individual in-depth interviews

For individual in-depth interviews, we recruited survey participants (stylists and clients) using purposive sampling to achieve a broad spectrum of perceptions while reaching thematic saturation. Based on data from the survey, we specifically invited stylists and clients with opposing views (e.g. not comfortable offering/receiving certain HIV/SRH services vs very comfortable offering/receiving certain HIV/SRH services) or who were critical about the project. All interested individuals were sent a digital copy of the consent form in their preferred language prior to the set interview date. The interview guide was developed collaboratively with the Citizen Scientist Working Group (Text S2). The aim was to explore five domains: i) perception of offering/receiving each of the 13 HIV/SRH services at hair salons (Text S6), ii) facilitators of offering/receiving any HIV/SRH service at hair salons, iii) barriers to offering/receiving any HIV/SRH service at hair salons, iv) general HIV/SRH knowledge, access, uptake and stigma, and v) the involvement in research as citizen scientists (among stylists only). The interviews were conducted in-person, at a confidential and comfortable space according to participants’ preferences. Participants were compensated for their time and travel costs between 50 to 100 LSL (2.9 to 5.8 USD), depending on their travel distance. All interviews were done in English or Sesotho, facilitated by a trained qualitative researcher, herself a young woman from Lesotho, fluent in both English and Sesotho, audio-recorded, and subsequently translated and transcribed into English. We collaboratively developed a rapid thematic analysis matrix [29] with pre-specified themes across the five above-mentioned overarching domains.

### Quantitative and qualitative sample sizes, data analyses, and triangulation

Sample size calculations followed corresponding guidance for cross-sectional cluster surveys [39] and were based on the only available quantitative evidence [20] indicating that 77% of clients were comfortable receiving PrEP at a hair salon in South Africa. Based on this, we calculated that a sample size of at least 300 clients across 100 stylists (with an intra-cluster correlation coefficient [ICC] of 0.05, a commonly recommended ICC for PrEP uptake [40, 41]) would be required to estimate the proportion of clients being comfortable receiving PrEP at hair salons in Lesotho with a 5% margin of error for a 95% confidence interval (details available on GitHub ). Following guidance for sample size in qualitative research [42], we planned 15 interviews or until reaching thematic saturation during the rapid thematic analysis.

The survey data was analyzed using appropriate descriptive statistics (frequency and interquartile ranges, mean/median and standard deviation). We stratified all client data analyses by age 15–24 years and above, and collapsed the 5-level likert scales throughout into three categories (i.e., combining “strongly agree” with “agree”, “strongly disagree” with “disagree”, and “neither agree nor disagree” as “neutral”). Data management and analysis was conducted using R version 4.3.3 [43].

The qualitative data analysis, following rapid thematic analysis approach guidance [29], was conducted in English and supported by Avidnote, an academic artificial intelligence tool for natural language processing with secured and regulatory-compliant data storage. We developed prompts for Avidnote to analyze each uploaded transcript to a) summarize the participants’ perception on each of the five domains (see above; last domain only among stylists) and b) pull illustrative quotes for each domain as well as for each of the individual 13 HIV/SRH services assessed in the survey. The results were fed into the pre-specified analysis matrix with deductive codes and additional codes were added inductively. This process was piloted with sample interviews and the prompts were refined after the first transcript that was in parallel independently analyzed manually by MK and MS to establish intercoder reliability.

We triangulated the qualitative data from domains (i) to (iii) with the quantitative data to achieve our aim of assessing acceptability and feasibility of offering/receiving HIV/SRH services at hair salons as comprehensively as possible. The data from domains (iv) and (v) will be reported elsewhere.

## Results

### Study participant characteristics

Between March and July, 2024, 157 stylists and 308 clients successfully registered, completed the questionnaire, and were eligible (flowchart in Figure S2). We recruited hair salons/stylists from all ten districts of the country, though most salons were located in urban and suburban areas with the majority (68.2%) concentrated in the capital Maseru (Figure S3). Stylists recruited a median of 3 clients (range: 1–5).

Most of the stylists identified as women (92.4%) with a median age of 29 years (interquartile range [IQR] 25, 33) (Table 1). All clients identified as women, had a median age of 26 years (IQR 22, 30), and 128 of 308 (41.6%) were 15–24 years old (subsequently termed “younger clients”). Secondary or higher education was completed by 82.2% of stylists, 91.4% of younger clients, and 86.6% of older clients. 6.2% of the younger clients were married and 42.5% of the older clients. Economically, only 10.9% of younger clients and 23.3% of older clients reported their financial situation being “comfortable” or “very comfortable”. 2.5%, 0.8%, and 1.1% reported living with HIV among the stylists, younger clients and older clients, respectively. Among stylists not living with HIV and reporting a self-perceived HIV acquisition risk, 25.6% indicated medium–high risk. This was the case for 19.6% of the younger and 29.9% of the older clients. The baseline characteristics of the 14 interviewees were representative of the survey participants and included survey participants with differing views about offering/receiving HIV/SRH services at the hair salon (Table S1).

**Table 1:** Stylists and clients'baseline characteristics

		**Stylists**	**Clients**		
		**Overall**	**Overall**	**15–24 yrs**	**25–35 yrs**
		n = 157	n = 308	n = 128	n = 180
Gender (%)	Woman/girl	145 (92.4)	308 (100.0)		
	Man/boy	12 (7.6)	0 (0.0)		
Age (median [IQR])		29.0 [25.0, 33.0]	26.0 [22.0, 30.0]	21.0 [20.0, 23.0]	29.0 [27.0, 32.0]
Education (%)	None	1 (0.6)	0 (0.0)	0 (0.0)	0 (0.0)
	Primary	8 (5.1)	10 (3.2)	3 (2.3)	7 (3.9)
	High school	67 (42.7)	146 (47.4)	77 (60.2)	69 (38.3)
	Vocational school	14 (8.9)	14 (4.5)	4 (3.1)	10 (5.6)
	Tertiary	62 (39.5)	127 (41.2)	40 (31.2)	87 (48.3)
	Prefer not to answer	5 (3.2)	11 (3.6)	4 (3.1)	7 (3.9)
Religion (%)	Christianity	144 (91.7)	286 (92.9)	117 (91.4)	169 (93.9)
	None	7 (4.5)	7 (2.3)	1 (0.8)	6 (3.3)
	Prefer not to answer	6 (3.8)	12 (3.9)	8 (6.2)	4 (2.2)
	Other	0 (0.0)	3 (1.0)	2 (1.6)	1 (0.6)
Owner of the hair salon (%)	Yes	129 (82.2)			
	No	28 (17.8)			
Working experience as a stylist (%)	Up to 1 year	28 (17.8)			
	1 to 3 years	51 (32.5)			
	3 to 6 years	27 (17.2)			
	More than 6 years	51 (32.5)			
Relationship status (%)	Single		205 (66.8)	114 (89.1)	91 (50.8)
	Married		84 (27.4)	8 (6.2)	76 (42.5)
	Cohabiting		5 (1.6)	4 (3.1)	1 (0.6)
	Separated		8 (2.6)	2 (1.6)	6 (3.4)
	Widowed		5 (1.6)	0 (0.0)	5 (2.8)
Number of children (%)	None		157 (51.0)	108 (84.4)	49 (27.2)
	1		101 (32.8)	17 (13.3)	84 (46.7)
	2		39 (12.7)	3 (2.3)	36 (20.0)
	3		11 (3.6)	0 (0.0)	11 (6.1)
	More than 3		0 (0.0)	0 (0.0)	0 (0.0)
Economical status (%)	Very poor		37 (12.0)	15 (11.7)	22 (12.2)
	Poor		53 (17.2)	26 (20.3)	27 (15.0)
	Getting by		124 (40.3)	53 (41.4)	71 (39.4)
	Comfortable		45 (14.6)	9 (7.0)	36 (20.0)
	Very comfortable		11 (3.6)	5 (3.9)	6 (3.3)
	Prefer not to answer		38 (12.3)	20 (15.6)	18 (10.0)
Occupation (%)	Employed		83 (26.9)	16 (12.5)	67 (37.2)
	Self-employed		60 (19.5)	14 (10.9)	46 (25.6)
	Homemaker		14 (4.5)	4 (3.1)	10 (5.6)
	Student		66 (21.4)	54 (42.2)	12 (6.7)
	None		66 (21.4)	29 (22.7)	37 (20.6)
	Prefer not to answer		19 (6.2)	11 (8.6)	8 (4.4)
Self-perceived HIV risk (%)	No risk at all	49 (31.2)	108 (35.1)	49 (38.3)	59 (32.8)
	Small risk	30 (19.1)	66 (21.4)	29 (22.7)	37 (20.6)
	Medium risk	25 (15.9)	51 (16.6)	18 (14.1)	33 (18.3)
	High risk	3 (1.9)	6 (1.9)	1 (0.8)	5 (2.8)
	Very high risk	1 (0.6)	3 (1.0)	0 (0.0)	3 (1.7)
	HIV already diagnosed	4 (2.5)	3 (1.0)	1 (0.8)	2 (1.1)
	I do not know	35 (22.3)	54 (17.5)	22 (17.2)	32 (17.8)
	Prefer not to answer	10 (6.4)	17 (5.5)	8 (6.2)	9 (5.0)

### PrEP, family planning, and menstrual health

Among stylists, 97.5% had heard of HIVST and 91.7% of PrEP; among clients, this was the case for 96.4% and 82.8%, respectively (Table S2). PrEP myths varied with 41.0% of stylists and 42.7% of clients supporting the statement that “PrEP will cause people to have more risky sex”. 63.1% of stylists reported currently using a contraceptive method (mainly male/external condom (32.3%) and the birth control pill (31.3%), most often obtained at a health facility), while 54.9% of clients reported currently using a contraceptive method (mainly male/external condom (45.0%), most often obtained at a health facility). Across all participants who experienced menstruation, disposable sanitary pads were the most commonly used menstrual health product (> 90%). 29.5% of stylists and 24.7% of clients mentioned having missed at least one day of school or work because of menstruation in the past year, mainly due to period pain.

### Acceptability of providing HIV/SRH services at hair salons

**Fig. 1:**
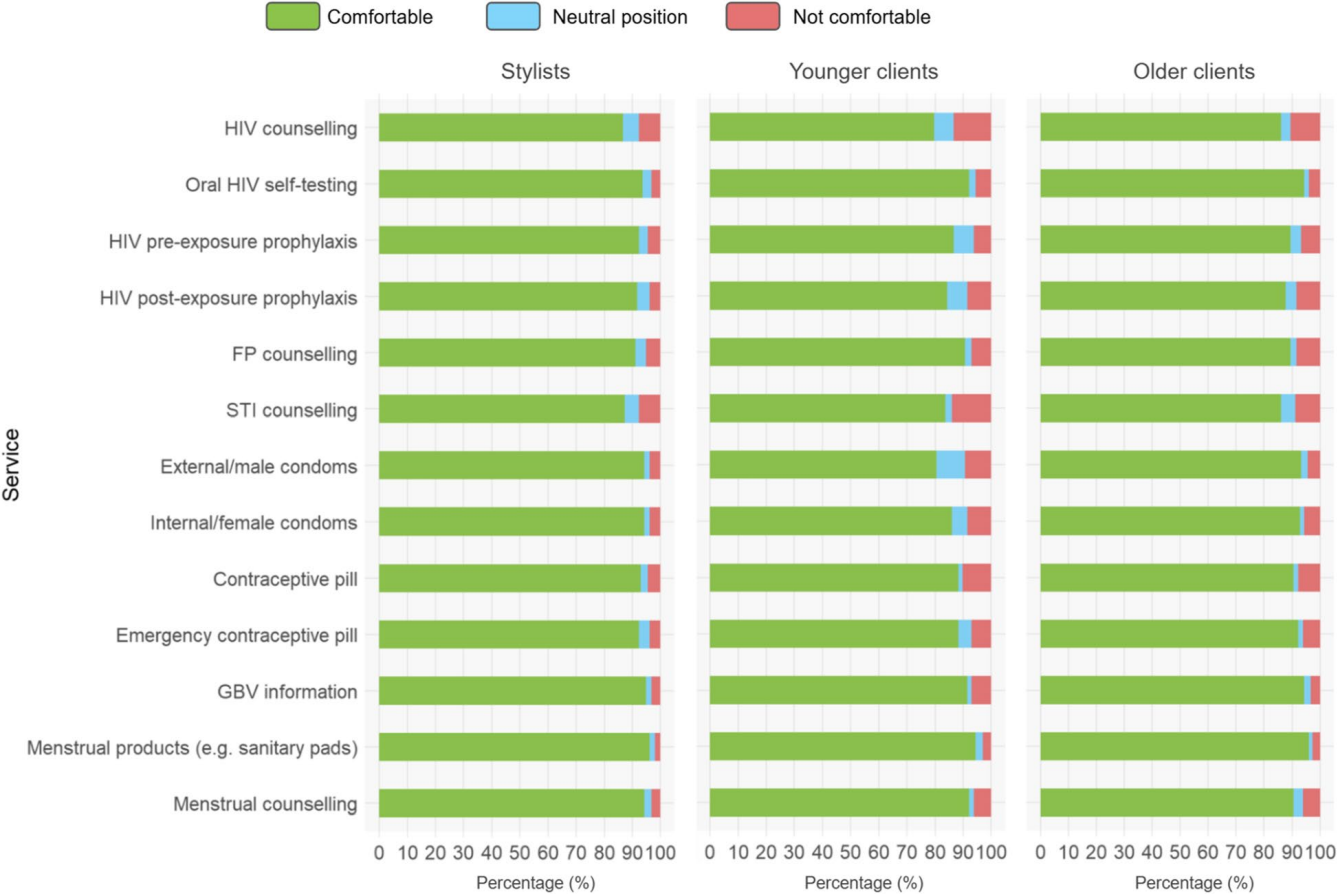
Comfortability of stylists to offer, younger clients and older clients to receive HIV/SRH services at hair salons in Lesotho. SRH: Sexual and Reproductive Health; FP: Family Planning STI: Sexually Transmitted Infection; GBV: Gender-Based Violence; MM: Menstrual Health

Among stylists, 86.6% (95% CI: 0.81, 0.91) agreed or strongly agreed to being comfortable offering HIV counseling (Fig. 1, Tables S3). Comfortability levels for HIVST, PrEP and PEP were 93.6% (95% CI: 0.90, 0.97), 92.4% (95% CI: 0.88, 0.97), and 91.7% (95% CI: 0.87, 0.96), respectively. For family planning services (counseling, external/male condoms, internal/female condoms, contraceptive pill, emergency contraceptive pill), providing GBV information, and menstrual health services (counseling, sanitary pad provision) the proportion of stylists being comfortable offering these services was generally higher than for HIV-related services, ranging up to 96.2% for menstrual products.

Among younger and older clients, comfortability levels showed a similar pattern, but were overall slightly lower than among stylists, with 83.4% (95% CI: 0.79, 0.88) for HIV counseling, 93.5% (95% CI: 0.91, 0.96) for HIVST, 88.3% (95% CI: 0.85, 0.92) for PrEP and 86.4% (95% CI: 0.83, 0.90) for PEP (Fig. 1, Table S4).

**Fig. 2:**
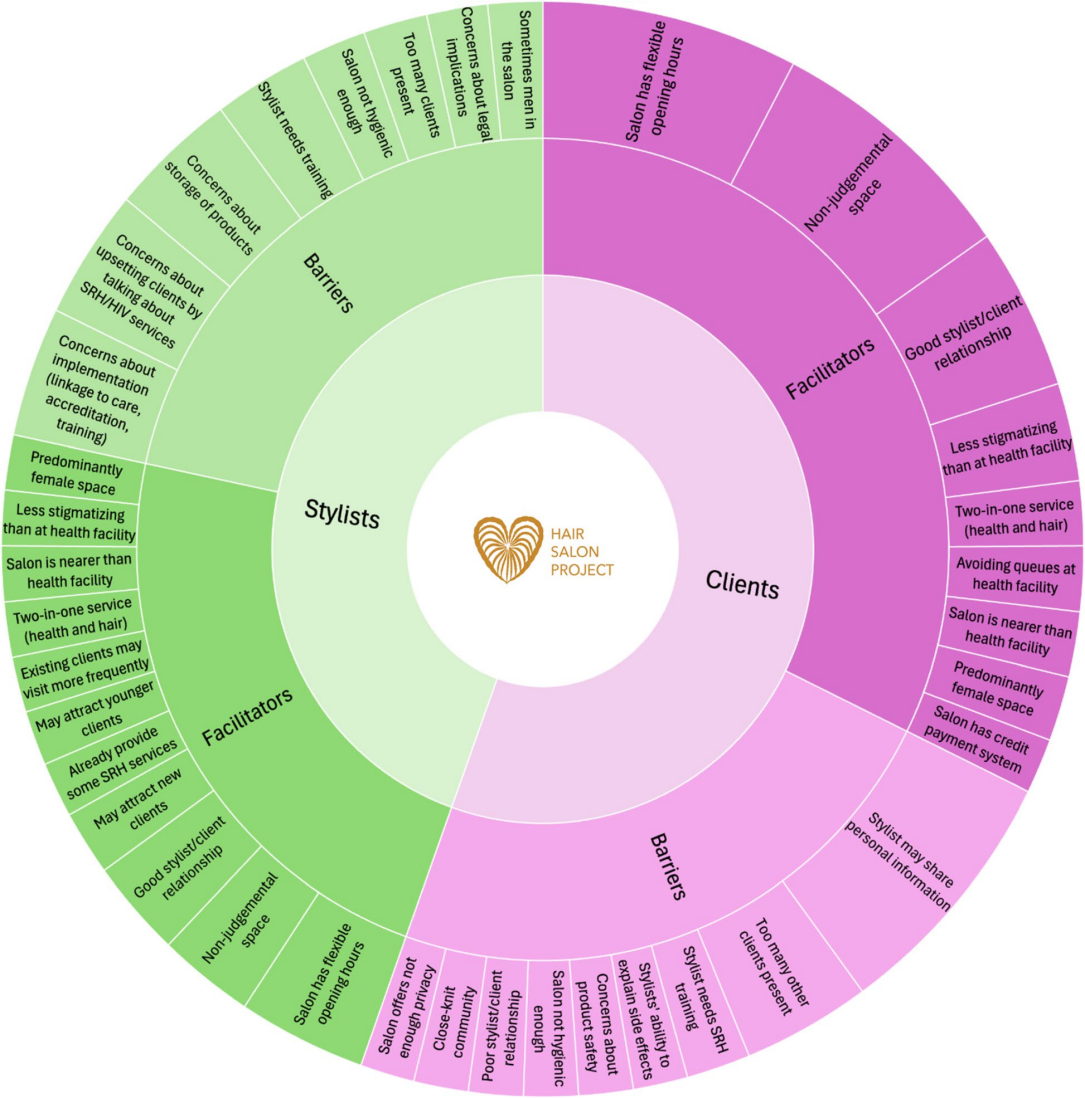
Barriers and Facilitators of offering/receiving HIV/SRH services at the hair salon. SRH: Sexual and Reproductive Health; The size of the codes (i.e., outer sunburst tiles) are according to how often these codes were mentioned by the interviewees

The interviews provided rich insights regarding barriers and facilitators of offering/receiving HIV/SRH services at hair salons. Figure 2 summarizes the codes and table S5 additionally the themes. Besides accessibility and convenience arguments outlined in the next section, stylists and clients mentioned themes like “safe space” and “non-judgemental” as facilitators of providing these services at hair salons:

*“I think another benefit can be that at salons, the stylists are able to talk to people so clients can be comfortable talking to them and getting help.”* [P2, client].

*“Yes, I think I’d be comfortable telling my clients that ‘here are some condoms, anyone who needs them can take them as needed”* [P1, stylist].

*“No, I wouldn’t have a problem [providing PEP] because we are also at risk, we could need it. Clients can also be at risk and need it.”* [P7, stylist].

Compared to HIV/SRH services offered at the clinic, many felt less judged at the hair salon:

*“A top challenge for me is that sometimes I don't want to go to the hospital. Some nurses ask questions that are too judgemental (laughs), yes we are young, and we are already sexually active, but the last thing we need is to be judged”* [P11, client].

*“Sometimes at the clinic, you are not free to talk about [SRH services]. You are being judged. You are talking to a stranger. […] When we are in the queues waiting for services, they specify which queue is for which service and one gets scared to queue on the right line because they believe everyone will see what they are at the clinic for. At the pharmacy too it's still a challenge, you check who is behind you to see if they will hear that you are there to buy condoms.”* [P3, stylist].

Some interviewees highlighted how integrating HIV services with SRH services as well as general beauty/hair services will facilitate and normalize acceptability of HIV services:

*“ I think since women already go to salons almost every month, it would be easier for them to get contraceptives from there. For example, if she uses monthly contraceptives, she can get two things done in one go: she will get her hair done, and get her contraceptives on the side.*” [P13, client].

Among the low number of stylists and clients who were uncomfortable providing/receiving HIV/SRH services at the hair salon, lack of privacy was the most common concern (Table S6 and S7). For services related to HIV, not feeling confident enough to provide them (among stylists) and preferring a healthcare professional instead (among clients) were the top concerns. Interviewees provided insights in this regard:

*“I think it's wise for me to attend the workshops about these services so as to have the correct information about them because I’m not a member of the health faculty. What I know is what I hear and what we tell one another. Therefore I think we should have professional training where we would have the correct information to pass on to our clients”* [P7, stylist].

*“I feel like things such as PrEP and HIV are very sensitive. You need someone who is very discrete, and someone with whom you will never have a conflict. I would much rather prefer to get it from the clinic”* [P11, client].

In addition to reported comfortability, we assessed actual interest in receiving SRH services at salons within the next six months (multiple answer options possible), HIV counseling was chosen by 14.6%, HIVST by 30.8%, PrEP by 22.1%, and PEP by 14.9% (Table 2). Oral HIVST was chosen similarly among younger and older clients, PrEP by 13.3% of the younger and 28.3% of older, and PEP by 7.0% of the younger and 20.6% of older clients. Family planning services, menstrual products and GBV information provision were chosen by more than a quarter overall, with no major differences between the two client groups. Only 2.9% of clients reported no interest in receiving any services at the salon.

**Table 2:** SRH services clients would be interested in the next six months, stratified by age group

Service	Clients		
	**Overall**	**15–24 yrs**	**25–35 yrs**
	**n = 308**	**n = 128**	**n = 180**
Family planning counselling	176 (57.1)	65 (50.8)	111 (61.7)
External/male condoms	75 (24.4)	27 (21.1)	48 (26.7)
Internal condoms/female condoms	70 (22.7)	21 (16.4)	49 (27.2)
Contraceptive pill	86 (27.9)	28 (21.9)	58 (32.2)
Emergency contraceptive pill	90 (29.2)	39 (30.5)	51 (28.3)
HIV counselling	45 (14.6)	16 (12.5)	29 (16.1)
Oral HIV self-testing	95 (30.8)	37 (28.9)	58 (32.2)
HIV pre-exposure prophylaxis	68 (22.1)	17 (13.3)	51 (28.3)
HIV post-exposure prophylaxis	46 (14.9)	9 (7.0)	37 (20.6)
Sexually transmitted infection counselling	54 (17.5)	16 (12.5)	38 (21.1)
Menstrual counselling	73 (23.7)	27 (21.1)	46 (25.6)
Menstrual products	94 (30.5)	40 (31.2)	54 (30.0)
Gender-based violence information	80 (26.0)	22 (17.2)	58 (32.2)
None	9 (2.9)	4 (3.1)	5 (2.8)

Participants could select more than one answer

A total of 10.8% of stylists believed that offering HIV/SRH services would negatively impact their business (Table S8):

“*Because some people may not be comfortable, and may feel like when they come to my salon, I assume things about them and their behaviors. […] Some may wonder ‘who is she to tell me about my sexual life?’* “ [P12, stylist].

Conversely, when stylists were asked if these services would have a positive impact on their business, 90.4% agreed, with the main reason being the potential to attract new clients (71.8%, Table S8). This was reflected among the stylist interviewees:

“*Well I think It’s going to bring clients in a sense that they know when they come to my salon they are not going to get services for the hair only […] I feel it's going to bring me more clients. One would tell the other that when you go to that salon you will also get this and this. So I think it is going to expose me to new clients.”* [P7, stylist].

One stylist also felt that more younger women would be willing to come to her salon as a result:

*“I think there will be more clients like youth. The youth are comfortable where they feel that they are not judged, and where they hear a person talking freely. Sometimes one can experience sexually transmitted diseases but isn't free to talk, but when they come to the salon they can be able to talk and get help.”* [P3, stylist].

### Feasibility of providing HIV/SRH services at hair salons

86.0% of the hair stylists reported that their hair salon is open seven days a week and 81.5% have a toilet on-site (Table 3). 60.5% mentioned that they are serving three or more clients simultaneously and only 21.0% have an additional confidential space—crucial aspects reflected upon in the interviews:

**Table 3:** Hair salon characteristics and feasibility measures

		**Stylists**	**Clients**
		**n = 157**	**n = 308**
Number of workers at the salon (%)	1–2	85 (54.1)	
	3–4	50 (31.8)	
	5 or more	22 (14.0)	
Simultaneous clients at the hair salon (%)	1–2	62 (39.5)	
	3–4	62 (39.5)	
	5 or more	33 (21.0)	
Number of clients per day (%)	1–3	69 (43.9)	
	4–6	60 (38.2)	
	7 or more	28 (17.8)	
The salon has a separate private room (%)	No	124 (79.0)	
	Yes	33 (21.0)	
The salon has a toilet (%)	No	29 (18.5)	
	Yes	128 (81.5)	
Salon’s opening hours (%)	Every day	135 (86.0)	
	Only in the evenings during weekdays	2 (1.3)	
	Only weekdays	16 (10.2)	
	Only weekends	4 (2.5)	
Frequency of hair salon visits (%)	More than twice a month		23 (7.5)
	Twice a month		86 (27.9)
	Once a month		174 (56.5)
	Once every two months		15 (4.9)
	Once every three months		6 (1.9)
	Less than once every three months		4 (1.3)
Duration of hair salon visits (%)	Less than an hour		29 (9.4)
	Between 1 and 2 h		133 (43.2)
	Between 2 and 4 h		123 (39.9)
	Between 4 and 6 h		17 (5.5)
	More than 6 h		6 (1.9)
Stylist would provide service if expenses are covered (%)	No	13 (8.3)	
	Yes	144 (91.7)	
Stylist would provide service if compensation is provided (%)	No	14 (8.9)	
	Yes	143 (91.1)	
Where would the stylist receive the material (%)	Somebody should bring it to the salon	78 (49.7%)	
	By themself at nearby health center	71 (45.2%)	
	By themself at nearby pharmacy	29 (18.5%)

*“You know! Sometimes there’s a random person sitting in the salon who isn’t even there to do her hair. I don't know, but I think of a cubicle in the same salon space which is enough for a private conversation. If only the client is comfortable moving to the private space to talk, and doesn’t mind that other clients will see her going to that private space. I agree that the salon space is not safe [in terms of privacy] because usually there’s a walk-in service where other people may just drop by and interrupt your conversation. So I think a small cubicle may be a good idea.”* [P1, stylist].

Concerning reimbursement expectations, 91.7% of stylists indicated they would provide HIV/SRH services even if their time is not compensated as long as the costs for the products and supply are covered (Table 3). The most favored supply option for stylists was having someone delivering the HIV/SRV products (49.7%) (Table 3).

56.5% of the clients visit their salon once a month and 35.4% even more frequently (Table 3). For 83.1% of clients, a hair salon visit lasts between 1 and 4 h. More than 82% of clients reach their hair salon within 30 min and 68.1% spend less than 20 Maloti (about $1.14 USD) on travel costs (Table S9). Hair salons seemed slightly more accessible than the nearest healthcare centers in terms of cost and time (Table S9), as mentioned in the interviews too:

*“It could benefit them because walking from my village to the health center is a long distance, so if one goes to a hair salon close by, it wouldn't take long like walking to the health center.”* [P2, client].

The qualitative data additionally emphasized the salons flexibility in terms of opening hours:

*“People usually go to the salons unlike to the clinic or to the pharmacy. Therefore it will be an advantage to my clients to get these services to the salon instead of going to the clinics. I also think the time at the salon is convenient for everyone. At the health centres, we go to on specific times and dates e.g. from Monday to Friday maybe 8 am to 4 pm. But at the salon, one goes every time.”* [P7, stylist].

“*is [the emergency contraceptive pill, i.e., plan B] the one that is used within a certain number of hours? […]. It would be better at the salon. At the clinic, I may need to wait in a long queue which would waste more time. But at the salon, I would just go in and get the plan B.”* [P11, client].

## Discussion

This innovative citizen scientist mixed-methods study among 157 hair stylists and 308 of their women clients aged 15–35 aimed to assess the acceptability and feasibility of offering/receiving various HIV/SRH services at hair salons in peri/urban areas across Lesotho. We found high acceptability with more than 83% of stylists and clients being comfortable offering/receiving services including HIV counseling, HIVST, PrEP, PEP, and over 90% of stylists thinking that this would have a positive impact on their business, attracting new and younger clients. Themes such as “safe space” and “non-judgemental” were commonly mentioned. Feasibility was equally high with salons being highly accessible, frequently visited and offering flexible opening hours. However, several concerns were raised, especially related to privacy and stylists’ role and capacity.

The only other published empirical evidence on the same topic from an African setting stems from three studies in South Africa [13–15] and one from Ghana [22]. However, the latter was merely a survey addressing SRH topics among hair stylists during the apprenticeship rather than providing services to their clients [22]. Similar to our study, a survey across 19 hair salons in the townships around Durban, South Africa, found that 98% of stylists felt comfortable offering health education if properly trained and the vast majority was comfortable with clients receiving injectable contraception (98%), PrEP (95%), and HIV testing (87%) provided by a nurse at their salon [20]. Among their women clients, 77% were comfortable receiving PrEP, and 74% HIV testing at the salon. In addition, the team from Durban interviewed > 90 clients and stylists, exploring specific services such as contraception and PrEP [19–21]. They identified similar facilitators to those observed in our study, such as the non-judgemental and high-trust nature of the client-stylist relationship, mutual investment of time at the salon and the high accessibility and conducive environment, all of which can support the delivery of community-based health services. Aligning with our findings, the concern most often mentioned was confidentiality. Importantly, the Durban studies are all based on a service model whereby a nurse is attached to the hair salon providing the services. While this offers certain advantages, it may have limitations in terms of sustainability and scalability. Our results suggest that a service model whereby the hair salon stylist is the main service provider reaches similar comfortability levels among both stylists and clients.

We observed substantial disparity between the comfortability levels and the more immediate demand. For example, 88.3% of clients were comfortable receiving PrEP at their hair salon from their stylist. However, when asked which of the 13 assessed HIV/SRH services they would opt for if provided at the hair salon in the coming 6 months, PrEP was chosen only by 22.1%. There are likely several explanations for this. First, not all surveyed clients may have been sexually active or some were already living with HIV or taking PrEP, and therefore should be removed from the denominator. Second, while we found that PrEP knowledge among clients was high (> 82%), myths around PrEP were prevalent. Third, not all clients at risk may also perceive themselves at risk, which is an important predictor and mediator for PrEP uptake [44–47]. Self-perceived high HIV risk among surveyed clients was low, especially among the younger clients, comparable to studies from similar settings [48]. Nonetheless, if the 22.1% PrEP demand translated into actual PrEP uptake, this would higher than at post abortion care clinics [49] and comparable to other community initiatives such as community health fairs [50], mobile teen health clinics [51] or pharmacy-based PrEP delivery [52].

The findings of this study are promising, however piloting an HIV/SRH service package at hair salons in Lesotho will provide a clearer understanding. Several recommendations for a pilot were drawn from the results. First, although the hair salon seems to be a trusted and non-judgemental space, privacy concerns were raised in case of actual service provision, especially for HIV services. Only a fifth of the salons reported a separate confidential space and most have several clients in parallel. Some interviewees suggested creating a confidential space within the hair salon (e.g., cubicles) either for confidential counselling or simply for confidential product retrieval. Second, the stylists’ role needs to be clarified and appropriate training ensured to capacitate the stylist. Third, one size may not fit all. The younger clients (15–24 years old) were markedly different in terms of sociodemographic characteristics (marital status, economic status, occupation) than the 25–35 years old clients, with more demand for HIV services among older than younger clients. This may partly be due to the fact that the HIV prevalence, and thus awareness, are higher among the older age group (28–40%) than among the younger age group (5–13%) [5]. However, the majority of clients across both age groups were comfortable with menstrual health and family planning services, signaled high demand for these services, and mentioned their role—besides the beauty/hair services—as facilitators for the HIV services. Fourth, the interaction between HIV/SRH service provision and the hair/beauty business needs to be explored. The majority of stylists thought that HIV/SRH services would positively impact their business by attracting new and potentially younger clients, and thus, agreed to provide them without additional compensation as long as they are appropriately trained, and supply is ensured. Fifth, more than half of the surveyed clients were already active users of different family planning methods. It remains to be seen how many new users of HIV/SRH services can be reached in this space. For long-term sustainability and based on results from a future pilot, a salon-based SRH program will require optimal integration and linkage with existing healthcare infrastructure (e.g. in terms of supply of products, supervision and referral), and a standardized training programme for stylists alongside continuous mentoring.

The strengths of this study are its nationwide sampling, the in-depth and innovative involvement of citizen scientists and the community, and its rigorous mixed-methods application for a more comprehensive perspective. However, there are limitations. First, due to our sampling approach, the majority of surveyed participants were from peri/urban areas with high levels of education, and thus, the results may not be applicable in rural areas. On the other hand, the density of new HIV transmissions in Lesotho is highest in these areas [53], where a future intervention might be most impactful. A future pilot will need to decide which population to target and adapt accordingly. Second, we lacked more in-depth information about sexual behavior, current and prior PrEP use. Third, HIV status was only self-reported indirectly through an HIV risk assessment question, likely leading to an underestimation of HIV prevalence. Fourth, stylists and clients who chose to participate may have been more open to the idea of salon-based health services, which may have introduced some social desirability bias and for example leading to a higher observed acceptability than in the general population. On the other hand, the questionnaires were completed in private. Fifth, for the interviews, we did not sample participants from all districts, due to budget constraints. Instead, and more importantly, we based our sampling strategy on reflecting different perceptions regarding service comfortability indicated in the survey.

## Conclusions

The findings of this nationwide, citizen-science mixed-methods study suggests that offering HIV/SRH services in hair salons in Lesotho seems to be largely acceptable and feasible if confidentiality concerns are mitigated. However, these results are solely based on survey data and not actual service provision. A pilot is warranted to investigate if acceptability can be translated into actual service uptake and to explore how this approach works in practice.

## Supplementary Information

Additional file 1.

## Data Availability

The datasets generated and/or analysed during the current study are available in the Zenodo repository, https://doi.org/10.5281/zenodo.14848731.

## Abbreviations

GRIPP2: Guidance for Reporting Involvement of Patients and the Public checklist
HIVST: HIV Self-Testing
MMR-RHS: Mixed Method Reporting in Rehabilitation & Health Services checklist
PEP: Post-Exposure Prophylaxis
PrEP: Pre-Exposure Prophylaxis
SRH: Sexual and Reproductive Health
WHO: World Health Organization
